# Cardiac Overload and Heart Failure Risk by NT-proBNP Levels in Older Adults with COPD Eligible for Single-Inhaler Triple Therapy: A Multicenter Longitudinal Study

**DOI:** 10.3390/jcm15010277

**Published:** 2025-12-30

**Authors:** Riccardo Sarzani, Francesco Spannella, Giorgia Laureti, Piero Giordano, Federico Giulietti, Alessandro Gezzi, Pier-Valerio Mari, Angelo Coppola, Roberta Galeazzi, Yuri Rosati, Erilda Kamberi, Andrea Stronati, Alessia Resedi, Matteo Landolfo

**Affiliations:** 1 Internal Medicine and Geriatrics, IRCCS INRCA, 60127 Ancona, AN, Italy; r.sarzani@univpm.it (R.S.); f.spannella@univpm.it (F.S.);; 2 Clinical and Molecular Sciences Department, “Politecnica delle Marche” University, 60126 Ancona, AN, Italy; 3 Internal Medicine, San Carlo di Nancy Hospital, 00165 Rome, RM, Italy; 4 Pulmonary Medicine, San Filippo Neri Hospital, 00135 Rome, RM, Italy; 5 Clinic of Laboratory and Precision Medicine, IRCCS INRCA, 60127 Ancona, AN, Italy; 6 Pneumology Unit, IRCCS INRCA, 60027 Osimo, AN, Italy; 7 Medicine Unit, AST Marche, 60025 Loreto, AN, Italy; 8 Pneumology Unit, AST Marche, 60035 Jesi, AN, Italy

**Keywords:** NT-proBNP, COPD, cardiac overload, heart failure, triple inhaled therapy, SITT

## Abstract

**Background:** In common clinical practice, cardiac overload is still often overlooked in patients with chronic obstructive pulmonary disease (COPD) despite its substantial impact on clinical outcomes and mortality. This study aimed to assess the prevalence of cardiac overload and heart failure (HF) risk, using N-terminal pro-B-type natriuretic peptide (NT-proBNP), in older COPD patients eligible for single-inhaler triple therapy (SITT) and without history of overt HF. We also evaluated changes in NT-proBNP after 3 months of SITT. **Methods:** This multicenter observational study included 165 older outpatients with a recent moderate-to-severe acute exacerbation of COPD (AECOPD), categorized as ‘Group E’ according to the Global Initiative for Chronic Obstructive Lung Disease (GOLD). Patients were stratified for the presence of cardiac overload and HF risk using age- and comorbidity-adjusted NT-proBNP thresholds, as recommended by the 2023 Clinical Consensus Statement of the Heart Failure Association (HFA) of the European Society of Cardiology (ESC). NT-proBNP was measured at baseline and after three months of SITT (116 patients with available test at three months). **Results:** Mean age was 80.7 ± 9.7 years. Patients with NT-proBNP levels indicative of “HF likely” and “HF very high-risk” were 43.0% and 24.2%, respectively. After 3 months of SITT, NT-proBNP significantly decreased by 7.2% (95%CI 9.0–5.4%, *p* < 0.001), with the largest reductions observed in younger patients [11.0% (95% CI 14.1–7.2%) ≤ 76 years old, 8.4% (95% CI −11.3–5.5%) in 77–87 years old, −3.0% (95% CI −6.1–0.0%) in ≥88 years old, p for interaction = 0.007]. **Conclusions:** In real-life clinical practice, a substantial proportion of older patients with GOLD Group E COPD had elevated NT-proBNP, suggestive of cardiac overload and high risk of HF. The early identification of these patients may prompt further cardiologic evaluation and management. After SITT and before cardiology evaluation, a significant NT-proBNP reduction has been observed, suggesting potential cardiovascular benefit of SITT.

## 1. Introduction

Chronic obstructive pulmonary disease (COPD) and its acute exacerbations (AECOPD) are major contributors to hospitalization, progressive loss of lung function, and reduced quality of life. These clinical burdens are compounded by underdiagnosed comorbidities—particularly cardiovascular (CV) and oncologic diseases—which significantly affect mortality [1]. COPD and CV disease share common risk factors and pathophysiological mechanisms, contributing to a higher prevalence of CV comorbidities in patients with COPD than in the general population [2]. CV disease has been identified as a leading cause of death in COPD patients [3,4,5], with heart failure (HF) showing the strongest association [6]. The coexistence of COPD and HF complicates the differential diagnosis of acute and chronic dyspnoea and influences the optimization of pharmacological treatments aimed at reducing hospitalization and mortality [7]. In the GULP study, chronic HF and ischemic heart disease were the only comorbidities independently associated with higher mortality in patients with COPD, regardless of airflow obstruction or hyperinflation severity [8]. This close and detrimental relationship underscores why early identification of cardiac overload is critical, as COPD can accelerate myocardial stress and dysfunction, leading to significantly increased morbidity and mortality in HF patients [9].

The failure to account for the close relationship between the lungs and the heart, both in guidelines and in clinical practice, could lead to a mismanagement of patients with COPD and HF [10,11].

While echocardiography is the gold standard for defining cardiac structure and function in the diagnosis of HF—and for classification according to left ventricular ejection fraction (EF)—its availability and feasibility may be limited in many clinical settings, especially among older populations and in non-cardiology settings [12]. Furthermore, in the context of advanced age and common comorbidities (including impaired renal function, alterations in body composition, and arrhythmias), the use of readily available biomarkers of cardiac overload, such as NT-proBNP, maintains a discriminative role in the diagnostic work-up of HF only at extreme values (rule-out or rule-in) [13]. Therefore, despite more than two decades of guidelines endorsement, NT-proBNP remains underused in clinical practice [14].

However, as endorsed by a recent Clinical Consensus by the European Society of Cardiology (ESC), the accurate use and interpretation of NT-proBNP cut-offs—after careful adjustment for key confounders such as age, body mass index (BMI), estimated glomerular filtration rate (eGFR), and atrial fibrillation (AF)—can also be regarded as a tool for the stratification of patients in terms of HF risk. This paves the way for NT-proBNP as a powerful screening tool to identify cases requiring echocardiography and further cardiological investigations [15].

Given these premises, and based on real-world clinical practice data, this study aimed to assess the prevalence of cardiac overload and HF risk, according to age- and comorbidity-adjusted NT-proBNP levels, in older COPD outpatients eligible for single-inhaler triple therapy (SITT) with no previous history of overt HF. As a secondary objective, we evaluated changes in NT-proBNP after three months of SITT in this same population.

## 2. Materials and Methods

### 2.1. Study Design and Population

This was an observational, multicenter, longitudinal study conducted from September 2022 to September 2024. Consecutive outpatients were enrolled across several Italian centers, including the Internal Medicine and Geriatrics (coordinating center) and Pneumology Units of IRCCS INRCA (Ancona and Osimo, Italy), Pneumology Units of AST Marche (Jesi and Loreto, Italy), Internal Medicine Unit of “San Carlo di Nancy” Hospital (Rome, Italy), and Pulmonary Medicine Unit of San Filippo Neri Hospital (Rome, Italy).

Eligible patients were aged ≥ 65 years with a prior COPD diagnosis and a clinical indication for single-inhaler triple therapy (SITT), according to the 2024 Global Initiative for Chronic Obstructive Lung Disease (GOLD) recommendations [11]. These criteria encompass patients with a history of at least one hospitalization due to AECOPD, or at least two moderate AECOPD per year (Group E). In this paper, AECOPD were confirmed through clinical records and referred to severe exacerbations that led to emergency room visits or hospitalization of the patient. Both treatment-naïve patients and those already taking inhaled therapy were included. All patients exhibited at least one respiratory symptom consistent with pulmonary or cardiac disease. We applied the following exclusion criteria: history of HF or primary cardiomyopathy, significant known valvular heart disease, asthma, end-stage renal disease (eGFR < 15 mL/min/1.73 m^2^) or dialysis, terminal conditions (e.g., advanced cancer, severe dementia, decompensated cirrhosis, bed rest syndrome), non-cardiac conditions potentially affecting NT-proBNP levels (e.g., acute kidney injury, sepsis), current triple inhaled therapy use before enrollment and patients unable to take inhalation therapy properly or unable to attend follow-up appointments. Given the observational nature of the study, the specific SITT combinations, among those commercially available in Italy, have been chosen by physicians according to daily clinical practice, regardless of whether or not the patient participated in the study.

Patients were assessed at baseline (T0) prior to SITT initiation and after 3 months (T3). For longitudinal analysis, patients who developed acute conditions during follow-up—such as infections, cardiac or pulmonary events, AECOPD, or new cardiac diagnoses—were excluded. At the same time, patients identified in the “HF very unlikely” category (see below) were excluded, because these patients had NT-proBNP levels indicative of no cardiac overload, and the search for any NT-proBNP decreases in this population holds no clinical significance (see flowchart in Supplemental Figure S1).

This investigation was conducted in accordance with the principles of the Declaration of Helsinki and its subsequent amendments. Written informed consent was obtained from all participants. The study protocol was approved by the local ethics committee (Comitato Etico, protocol code 22017, approval date 23 June 2022).

### 2.2. Clinical and Laboratory Assessments

Baseline anthropometric data and medical history were recorded. Obesity was defined as BMI ≥ 30 kg/m^2^. Blood samples were collected before initiating SITT and again at the 3-month follow-up, measuring hemoglobin, NT-proBNP, and serum creatinine.

Arterial hypertension was defined as blood pressure ≥ 140/90 mmHg (average of three seated measurements) or the use of antihypertensive medications. Dyslipidemia was defined by lipid-lowering therapy use or low-density lipoprotein cholesterol (LDL-C) levels exceeding guideline-recommended thresholds, adjusted for individual CV risk [16,17]. Chronic kidney disease (CKD) was defined as eGFR < 60 mL/min/1.73 m^2^ using the CKD-EPI formula. Smoking status was defined as current or former use of ≥100 lifetime cigarettes.

### 2.3. NT-proBNP Assay

NT-proBNP concentrations were measured using the Elecsys proBNPII electrochemiluminescence immunoassay on the Cobas e601 platform (Roche Diagnostics, Italy) [18]. This assay employs two monoclonal antibodies targeting the N-terminal region (amino acids 1–76) of proBNP.

### 2.4. Classification of HF Risk According to Age- and Comorbidities-Adjusted NT-proBNP

Patients were categorized according to the 2023 Clinical Consensus Statement of the HFA of the ESC [15], using age-specific and comorbidity-adjusted NT-proBNP cut-offs. Categories included were HF very unlikely, HF not likely (gray zone), HF likely, and HF very high-risk; each category received a specific recommendation in terms of further investigations to be addressed, from repeating the NT-proBNP assessment up to cardiology referral and echocardiography within 2 weeks (Table 1).

In accordance with this consensus, the age-adjusted NT-proBNP thresholds were further adjusted for specific comorbidities, following expected modifications of the biomarker levels in every clinical context:

- *Chronic kidney disease*: Given the proportional increment of NT-proBNP with the decline in renal function (eGFR), the threshold was increased by 15% for eGFR 45–60 mL/min/1.73 m^2^, by 25% for eGFR 30–45 mL/min/1.73 m^2^ and by 35% for eGFR < 30 mL/min/1.73 m^2^.
- *Atrial fibrillation*: The derangement of atrial electrical activity can itself increase NT-proBNP levels due to myocardial stretch; therefore, the threshold was increased by 100% for heart rate > 90 bpm and by 50% for heart rate ≤ 90 bpm.
- *Obesity*: Patients with higher BMI tend to have lower circulating NT-proBNP due to augmented clearance by adipose tissue (increased degradation); therefore, the threshold was decreased by a range of 25 to 40% based on the specific BMI class.

Combined adjustments were applied in patients with multiple comorbidities. For instance, an obese patient (BMI 32 kg/m^2^) with eGFR of 50 mL/min/1.73 m^2^ would have a net −10% adjustment (−25% + 15%).

For further details, please refer to the reference paper of A. Bayes-Genis et al. [15]. A systematic echocardiographic assessment, required for a characterization and phenotyping of HF (e.g., classification according to left ventricular ejection fraction), was not included in our analysis and goes beyond the scope of our work, which is based on “real-world” clinical practice, where echocardiography is not always immediately available [12].

### 2.5. Statistical Analysis

Data were analyzed using SPSS version 23 (IBM Corp. Armonk, NY, USA). Continuous variables were expressed as mean ± standard deviation (SD) or median and interquartile range (IQR) as appropriate. Categorical variables were reported as percentages. Group differences at baseline were assessed with ANOVA or Kruskal–Wallis tests (continuous variables) and chi-squared tests (categorical variables).

NT-proBNP values were natural log-transformed when required for normalization. A stepwise multivariate logistic regression was performed to identify independent predictors of higher NT-proBNP levels at baseline. Variables significant in univariate analyses were entered into the model using a forward selection approach (entry *p* = 0.05; removal *p* = 0.10). A sensitivity analysis was conducted using E-values to assess potential unmeasured confounding effects [19] (See Supplementary Materials).

For longitudinal comparisons between baseline and 3-month follow-up, paired *t*-tests, Wilcoxon rank-sum tests, McNemar’s test, and repeated measures ANCOVA were applied. The *p*-value for interaction was obtained using ANCOVA on the natural log-transformed NT-proBNP ratio to baseline. A *p*-value < 0.05 was considered statistically significant.

## 3. Results

### 3.1. Baseline Characteristics

A total of 165 patients were included at baseline (T0). The mean age was 80.7 ± 9.7 years, with a slight female predominance (52.1%). Arterial hypertension was the most common comorbidity (83.3%), followed by smoking history (78.9%) and obesity (28.3%). In the 30 days before enrollment, 51.5% of patients had experienced a moderate or severe AECOPD.

At baseline, 3.8% of patients were on long-acting muscarinic antagonists (LAMA), 40.0% were on dual therapy with LAMA/long-acting β2-agonists (LABA), and 16.7% were on LABA/inhaled corticosteroids (ICS).

**Figure 1:**
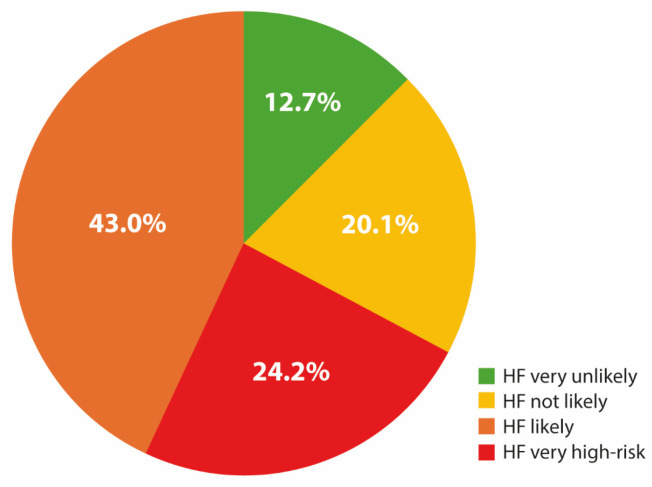
Distribution of patients across NT-proBNP-based “heart failure risk” categories according to age- and comorbidity-adjusted cut-offs.

The overall median NT-proBNP level before SITT initiation was 826.0 pg/mL (IQR: 262.0–2551.5). Based on age- and comorbidity-adjusted NT-proBNP cut-offs, 24.2% and 43.0% of patients fell into the “HF very high-risk” and “HF likely” categories, respectively. The remaining patients were categorized as “HF not likely” (20.1%) or “HF very unlikely” (12.7%) (Figure 1).

Patients with higher NT-proBNP levels were older and more likely to have experienced a recent AECOPD. They also had lower eGFR and hemoglobin levels, and were taking more antihypertensive medications (Table 2). Coronary artery disease, AF, and CKD were significantly more prevalent in these patients. Echocardiographic data were available in 55.8% (n = 92) of participants. The general characteristics of this subgroup are described in Supplemental Table S1. Among these, 59.0% had left ventricular hypertrophy (LVH), 60.8% had left atrial enlargement, and 23.1% had a left ventricular ejection fraction (LVEF) < 50%.

### 3.2. Predictors of Cardiac Overload or HF Risk

Multivariate logistic regression identified three independent predictors of falling into the “HF likely” or “HF very high-risk” categories (Table 3): AECOPD within the previous 30 days (OR 9.0, 95% CI 1.5–54.7; *p* = 0.017), lower hemoglobin levels (borderline significance, *p* = 0.057), and number of antihypertensive medications (OR 2.7, 95% CI 1.1–6.6; *p* = 0.031).

Sensitivity analysis using the E-value approach suggested that an unmeasured confounder would need to have a relative risk of at least 5.5 (lower bound: 1.3) to fully account for the observed association between recent AECOPD and “HF risk”.

### 3.3. NT-proBNP Changes After 3 Months of SITT

A longitudinal analysis was conducted on 116 patients before a cardiologic evaluation (Supplemental Table S2). The distribution of SITT formulations was as follows: 77.0% of patients on formoterol/glycopyrronium/budesonide 5/7.2/160 mcg (at the dose of two inhalations twice daily), 13.9% on fluticasone/umeclidinium/vilanterol 92/55/22 mcg (at the dose of one inhalation once daily), and 9.1% on beclomethasone/formoterol/glycopyrronium 87/5/9 mcg (at the dose of two inhalations twice daily).

**Figure 2:**
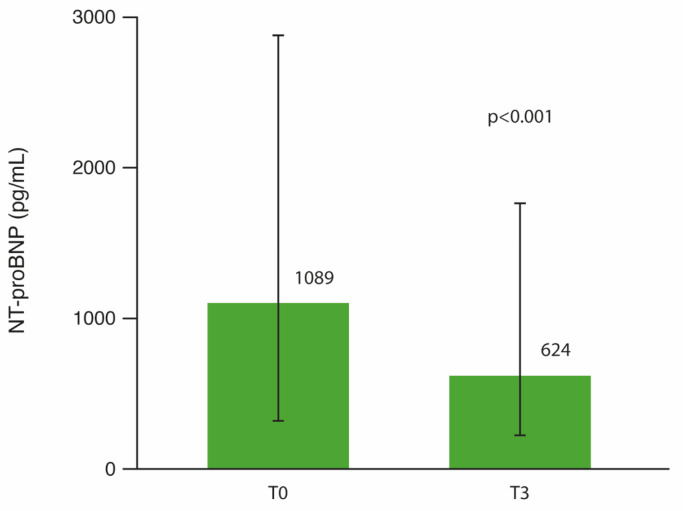
Change in median NT-proBNP levels before and after 3 months of single-inhaler triple therapy (SITT).

A significant reduction in median NT-proBNP values was observed at follow-up: 1088.5 pg/mL (IQR: 338.5–2876.5) at T0 vs. 624.0 pg/mL (IQR: 220.0–1716.3) at T3, *p* < 0.001 (Figure 2).

**Figure 3:**
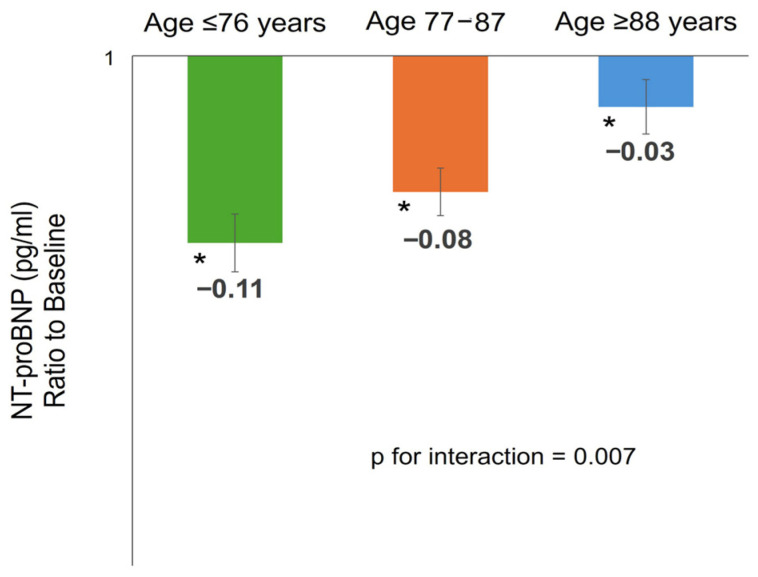
Log-transformed NT-proBNP change (ΔLn [NT-proBNP]) from baseline (T0) to 3-month follow-up (T3), stratified by age tertiles. * *p* < 0.05 for comparison between NT-proBNP at baseline and follow-up within the single subgroup. The p for interaction was obtained with ANCOVA of NT-proBNP ratio to baseline with baseline NT-proBNP as covariate.

After adjusting for baseline NT-proBNP levels, the mean relative reduction in NT-proBNP across the overall population was −7.2% (95% Confidence Interval [CI]: −5.4% to −9.0%; *p* < 0.001). No significant changes occurred in eGFR (49.6 ± 20.6 vs. 46.6 ± 20.0 mL/min/1.73 m^2^) or diastolic BP (70.5 ± 9.3 vs. 67.3 ± 10.0 mmHg; *p* = 0.058), but systolic BP decreased significantly (130.3 ± 15.6 vs. 122.4 ± 17.6 mmHg; *p* < 0.001). NT-proBNP reduction was inversely correlated with baseline NT-proBNP levels (p for interaction = 0.011). Sex (p for interaction = 0.155), obesity (p for interaction = 0.908), CKD (p for interaction = 0.787), or recent AECOPD (p for interaction = 0.238) did not significantly influence the reduction. Even after an exploratory sub-analysis, the type of SITT did not influence the trend in NT-proBNP (p for interaction = 0.732, Supplemental Figure S2). However, age had a significant interaction (p for interaction = 0.007), suggesting that the treatment effect varied based on patient’s age (Figure 3). The NT-proBNP reduction was most pronounced in the youngest tertile (age ≤ 76 years) at −11.0% (95% CI: −7.2% to −14.1%) and remained substantial in the middle tertile (age 77–87 years) at −8.4% (95% CI: −5.5% to −11.3%). Crucially, while a statistically significant reduction was still achieved in the oldest group (age ≥ 88 years), the magnitude of this effect was markedly attenuated, showing only a −3.0% change (95% CI: 0.0% to −6.1%).

### 3.4. Cardiovascular Therapy Adjustments During Follow-Up

Cardiovascular treatments were modestly changed during follow-up by investigators as per good clinical practice and before a specialist cardiology evaluation: renin–angiotensin–aldosterone system (RAAS) inhibitors increased from 67.5% to 75.0% (*p* = 0.453), β-blockers from 48.1% to 57.3% (*p* = 0.180), diuretics from 55.0% to 70.0% (*p* = 0.109). A gliflozin was initiated in 18 patients. NT-proBNP reduction remained significant even after excluding patients on gliflozin therapy [−6.1% (95% CI −10.0–−2.1%, *p* = 0.004)]. No significant interactions were observed between changes in CV therapy (p for interaction for RAAS inhibitors = 0.609, p for interaction for β-blockers = 0.342, p for interaction for diuretics = 0.412) and NT-proBNP at follow-up.

## 4. Discussion

In this multicenter study on older outpatients with COPD and no previous history of overt HF, nearly 70% had NT-proBNP levels indicating a likely or very high risk of underlying cardiac dysfunction. Independent predictors of this risk included a recent moderate or severe AECOPD, low hemoglobin levels, and the use of multiple antihypertensive medications, likely reflecting an increased burden of hypertension. Moreover, we observed a significant reduction in NT-proBNP levels after 3 months of single-inhaler triple therapy (SITT), particularly among younger patients, although a causal relationship cannot be established, given the observational nature of the study.

CV risk factors—especially arterial hypertension—and HF are highly prevalent in patients with COPD. HF should be considered not only a coexisting condition contributing to dyspnoea, but also a modifiable comorbidity that significantly impacts quality of life and clinical outcomes [20,21]. Previous studies have estimated HF prevalence in patients with established COPD to range between 7.1% and 31.3%, with a relative risk up to four times higher than in those without COPD [22]. Cardiac overload is likely present in many more patients, as shown by NT-proBNP levels in our sample, although patients with known HF were excluded from our analysis.

Multiple pathophysiological mechanisms contribute to the elevation of NT-proBNP levels in patients with COPD, reflecting underlying cardiac stress and dysfunction. Although not yet fully elucidated, these mechanisms include direct alterations of the left ventricle—such as myocardial fibrosis—leading to both mechanical and electrical impairment, alongside autonomic dysregulation, impaired ventricular filling, and increased afterload due to arterial stiffness [23]. The high prevalence of hypertension and other CV risk factors in the typically older COPD population further underscores the significant overlap between COPD and left-sided cardiac dysfunction. Additionally, structural remodeling of the pulmonary vasculature increases right ventricular afterload, thereby promoting the development of chronic *cor pulmonale* [23]. Findings from the Multi-Ethnic Study of Atherosclerosis (MESA) COPD Study demonstrated an independent association between residual lung volume and increased left ventricular mass, suggesting that COPD-related pulmonary hyperinflation contributes to elevated left ventricular wall stress [24]. Concurrently, hyperinflation in COPD and emphysema may decrease intrathoracic blood flow and preload, thereby impairing biventricular filling and reducing cardiac output [25]. Chronic hypoxemia and airway inflammation, mediated by the release of pro-inflammatory cytokines, may further promote myocardial inflammation and fibrosis, resulting in increased NT-proBNP release by cardiomyocytes. Notably, all these pathophysiological alterations are exacerbated during AECOPD. A meta-analysis confirmed significantly higher NT-proBNP levels in patients with COPD relative to non-COPD controls, with levels increasing in accordance with disease severity and further rising during AECOPD episodes [26]. In our cohort, a recent AECOPD (within the previous 30 days) emerged as an independent risk factor for high NT-proBNP levels. The EXACOS-CV ITALY study reported an approximately 34-fold increased risk of major CV events and a 51-fold increased risk of HF within the first 7 days following any exacerbation [27]. These findings were further validated by the PHARMO Data Network, which demonstrated that the elevated CV risk following moderate or severe AECOPD may persist for up to one year, though diminishing over time, with HF showing the most pronounced increase [28]. In this clinical context, NT-proBNP represents a valuable biomarker for assessing cardiac involvement in both stable COPD and during AECOPD episodes, exhibiting a strong prognostic utility in predicting mortality and future respiratory exacerbations [29,30,31].

Elevated NT-proBNP levels in older COPD patients may also reflect common comorbidities such as AF and CKD, although these parameters were not found to be independently associated in our sample. To address this, we applied comorbidity- and age-adjusted NT-proBNP cut-offs, in line with the 2023 Consensus of HFA of the ESC [15]. Using this refined approach, nearly 70% of patients in our real-world outpatient cohort met the threshold for cardiologic referral, with implications for early detection with cardiac ultrasound and management of cardiac overload.

Among other predictors, low hemoglobin and antihypertensive polypharmacy were independently associated with higher NT-proBNP levels. These may reflect a higher burden of systemic disease, impaired oxygen delivery, or long-standing hypertensive heart disease, which can exacerbate cardiac stress in the setting of COPD and advanced age [32,33].

Following SITT initiation, we observed a significant and consistent decline in NT-proBNP levels, suggesting a potential reduction in cardiac overload. The observational “real-world” design of the study does not allow us to make a causal link; however, NT-proBNP reduction was independent of changes in CV medications and main comorbidities. The observed reduction in NT-proBNP could stem from several interconnected mechanisms related to the components of SITT (ICS, LAMA, LABA) [3]. Triple therapy provides superior bronchodilation, leading to a marked decrease in pulmonary hyperinflation and intrathoracic pressure, which in turn improves biventricular filling, increases venous return, and reduces right ventricular afterload. Furthermore, by improving oxygenation and potentially reducing pulmonary vascular resistance, SITT may lessen the chronic strain on the right ventricle, thereby reducing NT-proBNP release from both ventricles. The ICS component of SITT could mitigate local inflammation and effectively reduce the rate of AECOPD, especially in high-risk patients like those in our GOLD E cohort. As our results show that a recent AECOPD is an independent predictor of high NT-proBNP levels, the sustained use of SITT inherently lowers the risk of acute exacerbation-related cardiovascular stress, contributing to a lower stable NT-proBNP level at the three-month follow-up. This is perhaps one of the most clinically relevant pathways by which SITT may confer cardiac benefit. The smaller reductions in NT-proBNP observed in older patients may reflect irreversible cardiac remodeling or fibrosis due to prolonged disease burden. This finding carries important clinical implications, suggesting that while SITT beneficially impacts cardiac overload markers across all ages, the therapeutic window for this specific effect may be smaller or less robust in the oldest old, who likely have more prolonged, fixed structural heart changes.

Our findings are also indirectly supported by the CLAIM study [34], which demonstrated improved cardiac preload and filling after dual bronchodilator therapy in COPD patients. The reduction in cardiac stress/overload in patients with severe COPD (GOLD E group) could play a key role in the prevention of CV events and sudden death, which are the most common causes of mortality in this population, also in light of the results of recent randomized trials such as ETHOS [35] and IMPACT [36], and metanalysis [37].

### Strengths and Limitations

This study is the first to apply highly specific NT-proBNP thresholds for HF risk that adjust not only for age—as commonly done in research and clinical practice—but also for critical comorbidities such as obesity, impaired renal function, and AF. This approach, aligned with the 2023 Clinical Consensus Statement of the HFA of the ESC, enhances the accuracy of our findings.

However, several limitations should be acknowledged. First, the sample size was relatively small, reflecting the strict inclusion criteria (i.e., older outpatients with GOLD E COPD and a clinical indication for SITT). Although statistically significant results were obtained, larger and more diverse cohorts are needed to confirm the generalizability of our findings. Second, we acknowledge the inherent risk of residual and unmeasured confounding in any observational study. Despite adjusting for a comprehensive set of variables strongly associated with NT-proBNP (including age, hypertension, obesity, and renal function), the influence of an unmeasured factor cannot be fully excluded (e.g., right heart strain or pulmonary hypertension). However, to mitigate this concern, our sensitivity analysis using the E-value method provides reassurance: it suggests that an unmeasured confounder would require a very strong association (risk ratio > 5.5) with both recent AECOPD and HF risk to entirely negate the observed relationship. We believe this E-value threshold makes it statistically improbable that a single unmeasured factor is solely responsible for our key findings. Third, a major limitation inherent to our real-world design is the lack of a randomized control group. This observational structure means that while we observed a significant association, we cannot establish a definitive causal link between SITT initiation and the reduction in NT-proBNP. We recognize that, given the ethical imperative to provide guideline-recommended therapy to these high-risk patients, withholding SITT would have been inappropriate. Therefore, the longitudinal component is best viewed as exploratory and hypothesis-generating. Future randomized controlled trials are essential to rigorously isolate the specific, causal impact of SITT on markers of cardiac stress. Fourthly, the exclusion of patients already on triple therapy and those with “HF very unlikely” from the longitudinal phase was guided by the study design and clinical rationale but may have created a selection bias. Lastly, echocardiographic data were not systematically available at both time points for all patients, reflecting real-world limitations in access to cardiac imaging—particularly in older populations. The characterization of cardiac morphology and the HF phenotyping through echocardiography were beyond the scope of our study, which focused instead on the use of age- and comorbidities-adjusted NT-proBNP cut-offs to assess the prevalence of cardiac overload and stratify the HF risk, thus identifying those patients to timely address to cardiac ultrasound imaging and cardiologic evaluation, as endorsed by the ESC Consensus Statement [15], which served as the foundation guide for our analysis. Another recent Clinical Consensus Statement pointed out how in the presence of signs/symptoms and markers of congestion (e.g., NT-proBNP), adequate management should not be delayed while waiting to receive an echocardiogram, as a long waiting time is often expected in clinical practice [12]. In agreement, being aware of the systematic absence of these echocardiographic parameters, we have always referred to “cardiac overload” or “HF risk” in relation to our data, throughout the paper.

## 5. Conclusions

This study underscores the clinical utility of age- and comorbidities-adjusted NT-proBNP as a low-cost, accessible biomarker to screen for the identification of cardiac involvement and HF risk in older patients with GOLD E COPD. Among individuals clinically eligible for single-inhaler triple therapy (SITT) without a previous history of known HF, nearly 70% of them exhibited NT-proBNP levels consistent with significant HF risk. The assessment of NT-proBNP plays a central role in the stratification and management of cardiopulmonary risk in COPD patients, as also stated by a recent international multidisciplinary consensus on this topic [38].

Early detection of cardiac stress in this population may facilitate timely cardiologic evaluation and targeted management of comorbid HF, potentially reducing hospitalization and mortality risk. In addition, we found a significant reduction in NT-proBNP levels after three months of SITT, leading to the hypothesis of its possible beneficial effects on cardiac load, which will require confirmation in future ad hoc studies.

These findings highlight the potential role of NT-proBNP in COPD phenotyping and risk stratification. Future prospective and interventional studies are warranted to confirm the prognostic implications of NT-proBNP-guided COPD management and the cardiopulmonary benefits of SITT in this high-risk group.

## Figures and Tables

**Table 1:** Age-adjusted NT-proBNP thresholds for heart failure risk stratification based on the 2023 HFA-ESC Clinical Consensus.

Heart Failure Risk According to the 2023 ESC/HFA Clinical Consensus	NT-proBNP Cut-Points	Recommendation
Heart failure very unlikely	≤125 pg/mL	Evaluation for a non-cardiac cause of symptoms
Heart failure not likely	Gray zone	Consider alternative diagnosis and elective echocardiography if clinical suspicion remains
Heart failure likely		Treat as appropriate; arrange for echocardiography within 6 weeks
<50 years old	≥125 pg/mL	
50–74 years old	≥250 pg/mL	
≥75 years old	≥500 pg/mL	
Heart failure very high-risk	≥2000 pg/mL	Priority echocardiography and evaluation by heart failure team within 2 weeks

NT-proBNP: N-terminal pro-B-type natriuretic peptide. Data taken from [15].

**Table 2:** Baseline characteristics of the study population stratified by NT-proBNP-defined heart failure risk categories.

Demographics, Anthropometrics and Comorbidities	Overall Population (n = 165)	HF Very Unlikely/Not Likely (n = 54)	HF Likely (n = 71)	HF Very High-Risk (n = 40)	*p*-Value
Age (years)	80.7 ± 9.7	76.0 ± 9.1	80.4 ± 9.3	87.8 ± 6.6	<0.001
Sex (male)	47.9%	63.0%	39.4%	42.5%	0.025
BMI (kg/m^2^)	26.8 ± 5.6	27.1 ± 6.4	26.7 ± 4.7	26.4 ± 6.1	0.877
Recent AECOPD (previous 30 days)	51.5%	9.3%	59.2%	95.0%	<0.001
T2DM	33.6%	32.4%	35.1%	32.4%	0.950
Hypertension	83.3%	71.4%	91.1%	85.3%	0.033
Dyslipidemia	63.9%	69.6%	56.3%	73.1%	0.288
Coronary artery disease	29.1%	13.2%	31.6%	41.0%	0.023
Known peripheral artery disease	29.2%	39.1%	29.8%	19.2%	0.308
Cerebrovascular disease	12.5%	16.7%	10.7%	11.8%	0.572
History of atrial fibrillation	37.8%	21.1%	37.9%	53.8%	0.012
Chronic kidney disease	50.7%	26.3%	56.7%	66.7%	0.001
Smoking status	78.9%	80.4%	81.8%	71.4%	0.455
Pack per year	25 (13–40.5)	18 (10–50)	25 (14–35)	30 (17.5–40)	0.781
Cardiovascular therapies					
RAASi	63.9%	45.5%	71.8%	68.2%	0.107
Calcium channel blockers	20.5%	27.3%	17.9%	18.2%	0.458
Diuretics	55.4%	36.4%	61.5%	63.6%	0.109
Other anti-hypertensives *	55.9%	41.3%	59.7%	68.6%	0.037
Number of anti-hypertensive drugs	2.0 ± 1.1	1.4 ± 1.3	2.2 ± 1.0	2.3 ± 0.9	0.018
Lipid-lowering drugs	48.2%	47.2%	52.1%	43.3%	0.745
Main laboratory and spirometry parameters					
eGFR (mL/min/1.73 m^2^)	59.8 ± 21.1	70.1 ± 18.1	57.7 ± 20.7	52.4 ± 21.1	0.001
NT-proBNP (pg/mL)	826 (262–2551.5)	168 (99–290.3)	1079 (580–1611)	4662 (3166.5–7327)	<0.001
Hemoglobin (g/dL)	12.1 ± 1.6	12.7 ± 1.5	12.0 ± 1.7	11.6 ± 1.5	0.010
Eosinophils (/mmc)	206.5 (144.3–287.5)	212 (155–279.5)	191 (120–241)	223.5 (167.5–315.8)	0.140
FEV1 (% predicted) **	60.1 ± 16.4	58.4 ± 17.4	62.1 ± 15.7	61.3 ± 15.7	0.644

HF: heart failure; BMI: body mass index; AECOPD: acute exacerbation of chronic obstructive pulmonary disease; T2DM: type 2 diabetes mellitus; RAASi: renin–angiotensin–aldosterone system inhibitors; eGFR: estimated glomerular filtration rate; NT-proBNP: N-terminal pro-B-type natriuretic peptide; FEV1: forced expiratory volume in 1 s. * β-blockers, α-blockers, mineralocorticoid receptor antagonists. ** A valid spirometry within 1 year was available in 83 patients.

**Table 3:** Independent predictors of elevated heart failure risk (composite of “HF likely” and “HF very high-risk”) identified by multivariate logistic regression *.

Variable	Wald	OR (95% CI)	*p*-Value
AECOPD within the previous 30 days	5.7	9.0 (1.5–54.7)	0.017
Hemoglobin (g/dL)	3.6	0.6 (0.3–1.0)	0.057
Number of anti-hypertensive drugs	5.7	2.7 (1.1–6.6)	0.031

* Stepwise logistic regression with the variables with a significant association at univariate analyses as independent variables (age, AECOPD, hemoglobin, number of anti-hypertensive drugs, coronary artery disease, history of atrial fibrillation, chronic kidney disease and eGFR). AECOPD: acute exacerbation of chronic obstructive pulmonary disease.

## Data Availability

The datasets used and analyzed during the current study are available from the corresponding author upon reasonable request.

## Supplementary Materials

The following supporting information can be downloaded at: https://www.mdpi.com/article/10.3390/jcm15010277/s1, Figure S1: The flow of participants in the longitudinal study; Figure S2: Log-transformed NT-proBNP change (ΔLn [NT-proBNP]) from baseline (T0) to 3-month follow-up (T3), stratified by SITT molecule combinations; Table S1: Baseline characteristics of the 92 patients with available echocardiography parameters (n = 92); Table S2: Baseline characteristics of the 116 patients evaluated at the 3-month follow-up (n = 116).
