# Supplementary material for: Cardiac Overload and Heart Failure Risk by NT-proBNP Levels in Older Adults with COPD Eligible for Single-Inhaler Triple Therapy: A Multicenter Longitudinal Study
Source: J Clin Med. 2025 Dec 30;15(1):277. doi: 10.3390/jcm15010277 (PMC12787263; doi:10.3390/jcm15010277)
Supplement: Supplementary file 1 [file jcm-15-00277-s001.zip › jcm-4017091-supplementary.pdf]

SUPPLEMENTARY MATERIALS

Sensitivity analysis for unmeasured factors

A sensitivity analysis for the potential effect of unmeasured factors was performed by the E-value methodology of VanderWeele and Ding [18]. This method estimates the minimum strength of association that would be required between both an unmeasured factor and the outcome (HF risk in our case) to overcome the statistically significant effect observed in a study where residual confounding is a potential problem. The calculation is derived from the relative risk obtained from an adjusted analysis in the observational study. For the current study, the adjusted ORs for the association between "AECOPD within the previous 30 days" and "composite of HF likely and HF very high-risk" were 9.0 (95% CI 1.1-54.7) (see Table 3 of the main article). In this case, the E-value for the point estimate is 5.5 and for the lower confidence interval limit is 1.3.

Thus, following the suggested language of VanderWeele and Ding, we found that the observed OR of 9.0 could be explained by an unmeasured factor, that was associated with both "AECOPD within the previous 30 days" and "composite of HF likely and HF very high-risk" by a risk ratio of 5.5 each, above and beyond the measured factor, but weaker confounding could not do so. The confidence interval could be moved to include the null by an unmeasured factor that was associated with both "AECOPD within the previous 30 days" and "composite of HF likely and HF very high-risk" by a risk ratio of 1.3 each, above and beyond the measured factor, but weaker confounding could not do so.

The E-value is a continuous measure and VanderWeele and Ding did not propose any threshold cutoff. In the current study, it seems implausible, or at least unlikely, to have an unmeasured factor with the association with both measured factor and HF risk, especially given that the main well known risk factors for HF have already been taken into consideration in the analysis (see Table 3 of the main article). Moreover, as cited by VanderWeele and Ding "In the context of biomedical and social sciences research, effect sizes ≥2- or 3-fold occasionally occur but are not particularly common; a

variable that affects both measured factor and outcome each by 2- or 3-fold would likely be even less common”. Then, in this case, the estimate is likely to be at least moderately robust.

SUPPLEMENTARY FIGURES

Supplemental Figure S1. The flow of participants in the longitudinal study.

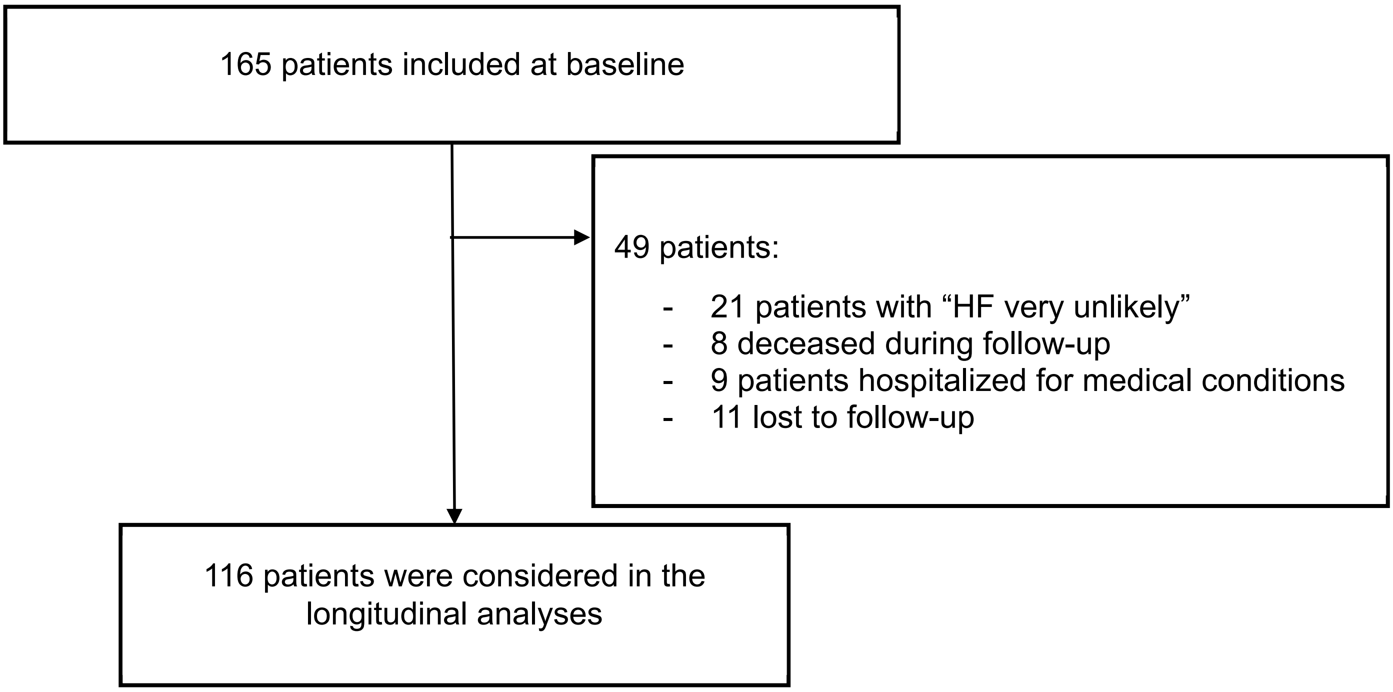

Supplemental Table S1. Baseline characteristics of the 92 patients with available echocardiography parameters (n=92).

**Demographics, anthropometrics and comorbidities**	
Age (years)	83.5±8.5
Sex (male)	43.4%
BMI (kg/m^2)	27.0±5.4
Recent AECOPD (previous 30 days)	30.3%
T2DM	33.3%
Hypertension	85.3%
Dyslipidemia	68.4%
Coronary artery disease	38.7%
Known peripheral artery disease	30.0%
Cerebrovascular disease	15.6%
History of atrial fibrillation	42.1%
Chronic kidney disease (eGFR <60 ml/min/1.73 m^2)	52.9%
Smoking status	76.7%
Pack per year	24.0 (11.0–35.0)
**Cardiovascular therapies**	
RAASi	72.5%
Calcium channel blockers	20.0%
Diuretics	57.5%
Other anti-hypertensives*	58.0%
Number of anti-hypertensive drugs	2.1±1.0
Lipid-lowering drugs	51.7%

**Main laboratory and spirometry parameters**	
eGFR (ml/min/1.73 m^2)	57.8±22.2
Hemoglobin (g/dL)	11.9±1.6
Eosinophils (/mmc)	117.0 (10.0–225.0)
FEV1 (% predicted)**	61.8±17.4

Data are presented as mean±SD, median (IQR) or percentage. HF: heart failure; BMI: body mass index; AECOPD: acute exacerbation of chronic obstructive pulmonary disease; T2DM: type 2 diabetes mellitus; RAASi: renin–angiotensin–aldosterone system inhibitors; eGFR: estimated glomerular filtration rate; FEV1: forced expiratory volume in 1 second.
*β-blockers, α-blockers, mineralocorticoid receptor antagonists.
**Available for 38 patients.

Supplemental Table S2. Baseline characteristics of the 116 patients evaluated at the 3-month follow-up (n=116).

**Demographics, anthropometrics and comorbidities**	
Age (years)	81.5±9.4
Sex (male)	46.6%
BMI (kg/m^2)	26.7±5.4
Recent AECOPD (previous 30 days)	55.2%
T2DM	31.5%
Hypertension	80.6%
Dyslipidemia	60.6%
Coronary artery disease	28.6%
Known peripheral artery disease	28.8%
Cerebrovascular disease	11.8%
History of atrial fibrillation	38.4%
Chronic kidney disease (eGFR <60 ml/min/1.73 m^2)	53.8%
Smoking status	77.0%
Pack per year	20.0 (11.0–40.0)
**Cardiovascular therapies**	
RAASi	67.5%
Calcium channel blockers	20.4%
Diuretics	55.0%
Other anti-hypertensives*	57.5%
Number of anti-hypertensive drugs	2.0±1.1
Lipid-lowering drugs	50.0%

**Main laboratory and spirometry parameters**	
eGFR (ml/min/1.73 m^2)	58.7±20.4
Hemoglobin (g/dL)	12.0±1.5
Eosinophils (/mmc)	207.0 (149.0–302.0)
FEV1 (% predicted)**	61.0±15.8

Data are presented as mean±SD, median (IQR) or percentage. HF: heart failure; BMI: body mass index; AECOPD: acute exacerbation of chronic obstructive pulmonary disease; T2DM: type 2 diabetes mellitus; RAASi: renin–angiotensin–aldosterone system inhibitors; eGFR: estimated glomerular filtration rate; FEV1: forced expiratory volume in 1 second.
*β-blockers, α-blockers, mineralocorticoid receptor antagonists.
**Available for 67 patients.

Supplemental Figure S2. Log-transformed NT-proBNP change (ΔLn [NT-proBNP]) from baseline (T0) to 3-month follow-up (T3), stratified by SITT molecule combinations.

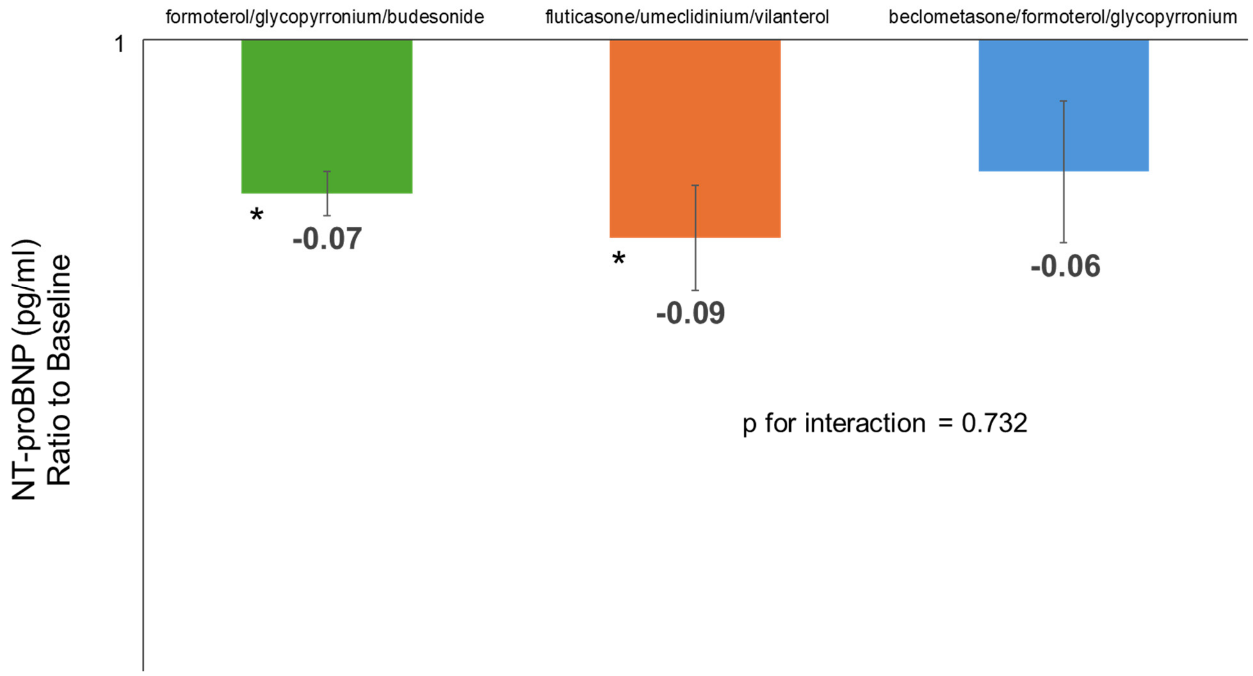

* p <0.05 for comparison between NT-proBNP at baseline and follow-up within the single subgroup. The p for interaction was obtained with ANCOVA of NT-proBNP ratio to baseline with baseline NT-proBNP as covariate. The ratio to baseline and the corresponding baseline value were ln-transformed before analysis to normalize their distributions.
